# Dispensable role of *Drosophila *ortholog of LRRK2 kinase activity in survival of dopaminergic neurons

**DOI:** 10.1186/1750-1326-3-3

**Published:** 2008-02-08

**Authors:** Danling Wang, Beisha Tang, Guohua Zhao, Qian Pan, Kun Xia, Rolf Bodmer, Zhuohua Zhang

**Affiliations:** 1 Burnham Institute for Medical Research, 10901 North Torrey Pines Road, La Jolla, California 92037, USA; 2 National Laboratory of Medical Genetics, Xiangya Hospital, Central South University, Changsha, Hunan, China; 3 Department of Neurology, Xiangya Hospital, Central South University, Changsha, Hunan 410078, China

## Abstract

**Background:**

Parkinson's disease (PD) is the most prevalent incurable neurodegenerative movement disorder. Mutations in *LRRK2 *are associated with both autosomal dominant familial and sporadic forms of PD. *LRRK2 *encodes a large putative serine/threonine kinase with GTPase activity. Increased LRRK2 kinase activity plays a critical role in pathogenic LRRK2 mutant-induced neurodegeneration *in vitro*. Little is known about the physiological function of LRRK2.

**Results:**

We have recently identified a *Drosophila *line with a P-element insertion in an ortholog gene of human *LRRK2 *(*dLRRK*). The insertion results in a truncated *Drosophila *LRRK variant with N-terminal 1290 amino acids but lacking C-terminal kinase domain. The homozygous mutant fly develops normally with normal life span as well as unchanged number and pattern of dopaminergic neurons. However, *dLRRK *mutant flies were selectively sensitive to hydrogen peroxide induced stress but not to paraquat, rotenone and β-mercaptoethanol induced stresses.

**Conclusion:**

Our results indicate that inactivation of *d*LRRK kinase activity is not essential for fly development and suggest that inhibition of LRRK activity may serve as a potential treatment of PD. However, *d*LRRK kinase activity likely plays a role in protecting against oxidative stress.

## Background

Parkinson's disease (PD) is a common and currently incurable neurodegenerative movement disorder affecting approximately 1–2% of the population over 65 years of age. Clinically, it is characterized by age-dependent resting tremor, muscular rigidity, and akinesia. Neuropathologically, selective loss of dopaminergic (DA) neurons in the substantia nigra compacta region and Lewy body formation in the remaining neurons are two hallmarks of PD patient brains [1].

The molecular mechanism of PD-specific neuropathological changes and parkinsonism motor deficits are largely unknown. Nevertheless, significant progress on molecular genetics of PD has been made during the last several years by studying familial PD cases. Mutations in at least 7 genes have been implicated in various forms of familial PD cases. These genes include α-synuclein, uchL1, LRRK2, parkin, PINK1, DJ-1, and ATP13A2 [2-10].

*LRRK2 *was recently identified as a novel gene responsible for an autosomal dominant form of PD, suggesting a toxic gain of function of LRRK2 in affected cases [3,5]. So far, at least 20 *LRRK2 *mutations have been identified from PD patients, accounting for ~7% familial form of PD cases and for a significant portion of sporadic PD cases [11,12]. Unlike other PD-associated genes, which normally are correlated with early-onset or pathologically atypical forms of PD, *LRRK2 *is associated with late-onset and clinically idiopathic PD [3,5,12]. Thus, dysfunction of LRRK2 may impair a common pathway involving in pathogenesis of both familial and sporadic PD cases.

LRRK2 is a large protein (2527 amino acids) consisting of several independent domains, including a leucine-rich repeat domain, a Roc GTPase domain followed by its associated C terminal of Roc (Rac) domain, a protein kinase domain of the MAPKKK family, and a C-terminal WD40 domain [13,14], suggesting a complexity of its cellular function and regulation. Recent studies suggest that LRRK2 can self-phosphorylate *in vitro*. Moreover, the kinase activity of LRRK2 seems to be tightly regulated by its GTPase activity [15]. PD related mutations results in increased kinase activity of LRRK2 [16,17]. Thus, inactivation of LRRK2 kinase activity constitutes a potential strategy for PD treatment. A critical point for this treatment strategy is whether inhibition of LRRK2 physiological activity will affect the normal development process or induce severe pathological side effects.

In the present study, we investigated roles of LRRK2 in development and neuronal survival using *Drosophila *as a model system. Our results suggest that LRRK2 kinase activity is not required for development, survival of DA neurons, and protection of PD-related stress of *Drosophila*.

## Results

### Identification of *Drosophila *Line with *LRRK2 *Deletion

Sequence analysis revealed a single *Drosophila *ortholog (CG5483) of human *LRRK1 *(*h*LRRK1) and *LRRK2 *(*h*LRRK2) [designated as *Drosophila LRRK *(*dLRRK*)]. *d*LRRK shares 24% identity and 38% similarity at the amino acid (aa) level to *h*LRRK2. The kinase domain is 31% identical and 52% similar between *d*LRRK and *h*LRRK2. The predicted critical amino acids for function of LRRK2, including proton acceptor (D1994), ATP binding site (K1906), and 9 of total 18 identified pathogenic mutant amino acids, are highly conserved [see Additional file 1] [12]. These results suggest that CG5483 is a *Drosophila *ortholog of both *h*LRRK1 and *h*LRRK2.

**Figure 1:**
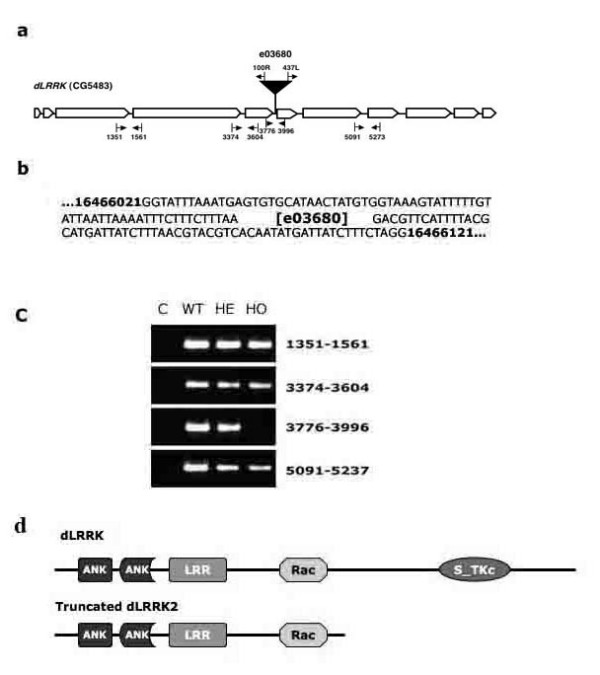
**Identification of *Drosophila *Line with *LRRK2 *Deletion**. Schematic representation of the piggyBac element insertion site of Drosophila line e03680. (a). The *dLRRK *gene has 11 exons (arrow boxes) and 10 introns (black line between exons). The piggyBac insertion site is indicted by a filled block arrow. 4 pairs of primers used for RT-PCR analysis of *dLRRK *transcripts are also indicated. These include 1351 and 1561; 3374 and 3604; 3776 and 3996; 5091 and 4237. Positions and directions of different primers are marked with vertical black lines and horizontal arrows. (b) *dLRRK *transcripts in various fly lines are detected with RT-PCR. C, negative control; WT, +/+ fly; HE, e03680/+ fly; HO, e03680/e03680 fly. (c). The flanking sequence of piggyBac insertion was shown. (d). Schematic representation of wt *d*LRRK protein and mutant *d*LRRK protein in fly line e03680. ANK, N-terminal ankyrin repeat domain; LRR, leucine-rich repeat domain; Rac, C terminal of Roc GTPase domain; S_TKc, Serine/Threonine protein kinases domain.

We identified a fly line (e03680) with piggyBac element insertion in the intron between exon 5 and exon 6 of *dLRRK *gene [18] (Fig. 1). RT-PCR detects a mutant *d*LRRK transcript with deletion of exon 6 (Fig 1a, b, c). Primer extension analysis revealed the mutant *d*LRRK transcript encoding N-terminal 1289aa, RYCNECA encoded by the intron between exon 5 and exon 6, followed by an in-frame stop codon. The transcript returns to exon 7 and the whole C-terminal sequence after exon 7. Therefore, the mutant fly harbors a truncated protein consisting of the N-terminal ankyrin repeat (ANK), leucine-rich repeat (LRR) and Rac domains. Thus, the resulted mutant fly carries a kinase-null *d*LRRK (Fig. 1d).

### *Drosophila *lacking *dLRRK *kinase activity are viable with normal development

**Figure 2:**
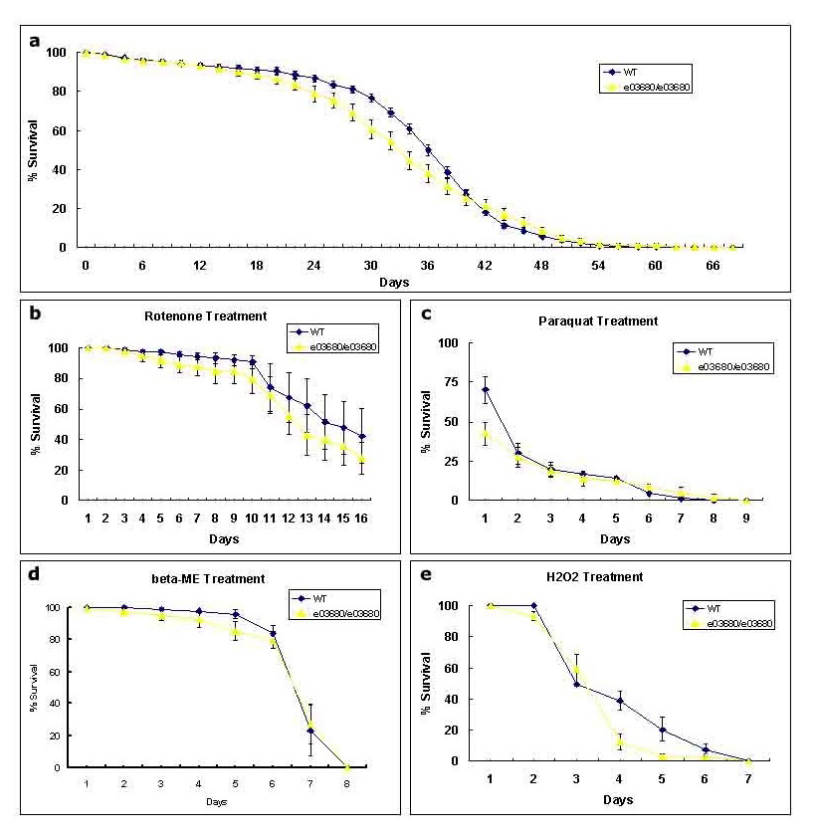
***dLRRK *mutant flies are selectively sensitive to hydrogen peroxide**. Wild type flies and *dLRRK *mutant flies have similar life span (a). Wild type flies and *dLRRK *mutant flies were treated with 250 μM rotenone (b), 5 mM paraquat (c), 2 mM β-ME (d) and 1% H_2_O_2 _(e). Note: *dLRRK *mutant flies have similar sensitivity to normal flies towards treatments of PD-associated stress inducer rotenone and paraquat, as well as misfolded protein stressor β-ME. *dLRRK *mutant flies show increased sensitivity to general oxidative stress inducer H_2_O_2_.

**Figure 3:**
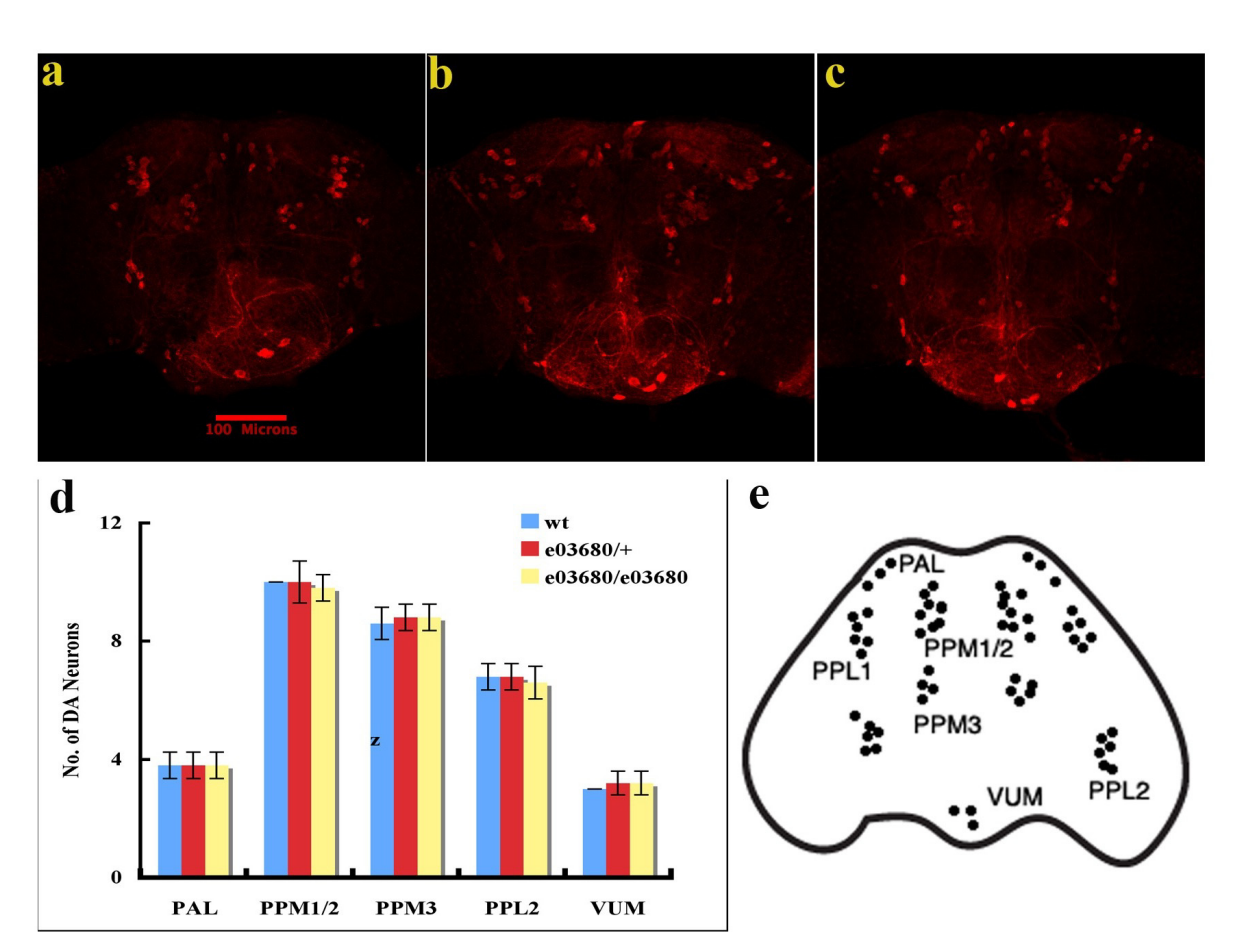
***Drosophila *lacking *dLRRK *kinase activity has normal development of DA neuron**. Brain dissected from wild-type flies (a), e03680/+ flies (b) and e03680/e03680 flies (c) aged 20 days were immunostained with anti-*Drosophila *TH antibody followed by an Alexa Fluor 594-labeled secondary antibody to indentify DA neurons. Representative pictures shown were collected by confocal microscopy. Dopaminergic neurons in six brain regions, including PAL, PPM1/2, PPM3, PPL1, PPL2, and VUM, were quantified and no significant difference was found among different fly lines (d). Localization of *Drosophila *DA neurons is illustrated in e.

Recent studies suggest that increased kinase activity plays critical roles in neuronal death induced by pathogenic *LRRK2 *mutants [17]. We therefore determined the roles of the *d*LRRK kinase activity in fly development and DA neuron survival. Homozygous *dLRRK *mutant files were generated. These mutant flies were viable, fertile and developed with no obvious preadult or afteradult external abnormality (not shown). They showed similar life span to their wildtype counterparts (Fig 2a). Whole-mount brain immunostaining with an anti-*Drosophila *tyrosine hydroxylase antibody revealed no detectable change of number and distribution of DA neurons in flies at age 20 days in comparison with wildtype control flies (Fig 3). The results suggest that inactivation of *d*LRRK kinase has little affect on development, life span, and survival of DA neurons in fly.

### *dLRRK *mutant flies are selectively sensitive to hydrogen peroxide

Oxidative stress is implicated in PD pathogenesis [19,20]. We next determined the effects of oxidative stress on *d*LRRK mutant flies. *d*LRRK mutant flies showed little difference in survival from their wiltype counterparts after exposure to oxidants rotenone (250 μM), paraquat (5 mM), and unfolded protein inducer β-mercaptoethanol (β-ME, 2 mM) (Fig. 2b, c, d) [21]. Paraquat and rotenone, mitochondrial complex I inhibitors, induce PD-like selective degeneration of DA neurons [22,23]. In contrast, hydrogen peroxide (H_2_O_2_) treatment (1%) resulted in increased death of *dLRRK *mutant flies comparing to their wildtype counterpart (Fig. 2e). Unlike paraquat and rotenone, H_2_O_2 _induces a more general oxidative stress that is not selective for the DA neuron. The results suggest that loss of LRRK function unlikely trigger the selective sensitivity to DA neuron preferential or mitochondria initiated oxidative stress in fly. However, *dLRRK *mutant flies are more sensitive to general and overall oxidative stress than wildtype flies.

## Discussion

*LRRK2 *mutations are linked to a significant number of both familial and sporadic PD cases, little is known about the biological functions and PD-related pathogenic mechanism of this protein. We have shown in this study that inactivation of *d*LRRK kinase activity has no effect on the development and DA neuronal survival of *Drosophila*. Recent studies using transfected cells suggest that PD-associated LRRK2 mutants have increased kianse activity. Moreover, the increased kinase activity is correlated with increased susceptibility to cell death [24]. This observation is consistent with finding of association of human *LRRK2 *mutations with autosomal dominant form of PD. The PD-associated LRRK2 mutant proteins likely contribute to PD pathogenesis via gain of deleterious functions. If LRRK2 functions are conserved between *Drosophila *and human, our observation suggests a potential strategy for PD treatment via developing LRRK2 kinase inhibitors

Another interesting finding of this study is that loss of LRRK2 kinase activity does not change sensitivity of *Drosophila *to PD related stress reagents. Rotenone and paraquat are shown to induce parkinsonism in multiple animal models and in human. Despite the precise mechanism remains unknown, the two chemicals inhibit mitochondrial complex I activity [25,26]. On the other hand, inactivation of *d*LRRK kinase activity results in increased susceptibility of *Drosophila *to a general oxidant H_2_O_2_. Together, these results suggest that *d*LRRK likely plays a role in protecting against non-mitochondrial oxidative stress. Nevertheless, the implication of this finding in PD pathogenesis needs to be further verified in mammalian models, given that there are two LRRK homologs in mammals.

A recent study suggests that inactivation of *d*LRRK results in severely impaired locomotive activity and degeneration of dopaminergic neurons [27] that are not found in this study. The discrepancy between the two studies remains to be resolved by further investigation.

## Conclusion

In summary, we have found that *d*LRRK kinase activity is not required for normal development and growth. The results will facilitate our understanding of pathophysiological function of human LRRK2.

## Methods

### *Drosophila *stocks and reagents

e03680 flies were obtained from the Exelixis collection at Harvard Medical School.  *Drosophila *were maintained on standard cornmeal-molasses-agar medium at 25°C.

Anti-*Drosophila *TH antibody (1:500) was generously provided by Dr. Neckameyer (Department of Pharmacological and Physiological Science, Saint Louis University School of Medicine, St. Louis, Missouri 63104), Alexa Fluor 594 goat anti-rabbit IgG was from Invitrogen (San Diego, CA). Hydrogen peroxide, paraquat, rotenone and β-ME were purchased from Sigma.

### Checking and Identification of piggyBac Insertion sites

Genomic DNA was purified from wild type, e03680/+ or e03680/e03680 flies. According to the piggyBac element insertion site information from Exelixis collection at Harvard Medical School. Several pairs of primers were designed around insertion site and PCR was performed to check the band size, including 1351–1561, 3374–3604, 3776–3996, and 5091–5237. For sequencing flanking sequence, we used primers 3776-100R and 473L-3996 to amplify 5' and 3' flanking DNA, then sent DNA fragment for sequencing. The sequences of primers are 1351: GTAAGGGTTCCCTGGATGGT; 1561: GGCCTATTGGTGCAGGTAGA; 3374: TAAGTTGCCGGACCCTACAC; 3604: 111TCATCTGTTCGGTGACCAAG; 3776: AGATCAACCCCTTTGCTCCT; 3995: AGCTTAACCGTGCTTCCTGA; 5091: AGGTGCTTTTGGGTTCGTTT; 5273: ATCCCGACCAAGGGTACAAT; 100R: TCCTAAATGCACAGCGACGG; 473L: ACCTCGATATACAGACCG.

Primers used for 5' RACE experiment including: RACE-1: GACTCGAGTCGACGAATTCAATTTTTTTTTTTTTTTTT; RACE-2: GACTCGAGTCGACGAATTCAA; 4042R: CGGACGGGAAATAAGTCATC; 3954R: CGAAGGCAGTAAGGAGGGTA

### Whole mount immunostaining

Whole mount immunostaining of fly brains was done as previous described [28]. Briefly, fly heads were fixed with 4% paraformaldehyde containing 0.2% Triton X-100 overnight and washed with PBT (PBS containing 0.2% Triton X-100) 3 times. Brains were dissected in blocking buffer (PBS, 5% heat inactivated normal goat serum, 0.2% Triton X-100), followed by blocking at room temperature for 1 hour. Brains were immunostained with corresponding primary antibodies at 4°C overnight followed by respective secondary antibodies at room temperature for 3 hr. DA neurons were quantified using confocal images and analyzed statistically using InStat 3 (GraphPad, San Diego).

### Lifespan

Approximately 500 flies per genotype were analyzed in the lifespan study. The flies were transferred to new vials every second day and the number of dead flies in each vial was recorded. The experiment was continued until all flies were dead. The percentage of flies alive at each time point was quantified and graphed.

### Compounds treatment

3–5 days old flies were used for all treatments. At least 100 flies were used for each treatment. Flies were first starved for 3 hours and then transferred to vials with filter papers soaked with toxic compound containing 5% sucrose. Flies were transferred to new vials with fresh compound every day, and the number of dead flies in each vial was recorded. The experiment was continued until all flies were dead. The percentage of flies alive at each time point was quantified and graphed. Chemical compounds were administered at the following doses: 250 μM rotenone, 5 mM paraquat and 2 mM β-ME.

## Abbreviations

Parkinson's disease (PD), dopaminergic (DA), human *LRRK2 *(*h*LRRK2), *Drosophila LRRK *(*dLRRK*), leucine-rich repeat (LRR), N-terminal ankyrin repeat (ANK), β-mercaptoethanol (β-ME), hydrogen peroxide (H_2_O_2_)

## Competing interests

The author(s) declare that they have no competing interests.

## Authors' contributions

DW carried out the molecular genetic studies to characterize *d*LRRK deletion line used in this study, did whole mount immunostaining and drafted the manuscript. GZ did the life span and stress treatments. BT, QP, KX, RB participated in experimental designs. ZZ designed the whole project and draft the manuscript.

All authors above have read and approved the final manuscript.

## Supplementary Material

Additional file 1 Amino acid sequence alignment of *h*LRRK2 and *d*LRRK. The data provided the amino acid sequence alignment of *h*LRRK2 and *d*LRRK. *h*LRRK2 amino acid sequences (NP_940980) and *d*LRRK amino acid sequences (CG5483, NP_650903) are from NCBI protein database. Alignment was done with MacVector™ 7.2.2. "*" Indicates identical amino acids between the two sequences. "." Indicates conserved amino acids between the two sequences. Highlighted purple indicates Roc GTPase domain. Highlighted red indicates kinase domain. Highlighted blue indicates the conserved PD associated point mutation sites between human and *Drosophila*. Click here for file
