# Supplementary material for: Dispensable role of Drosophila ortholog of LRRK2 kinase activity in survival of dopaminergic neurons
Source: Mol Neurodegener. 2008 Feb 8;3:3. doi: 10.1186/1750-1326-3-3 (PMC2276501; doi:10.1186/1750-1326-3-3)
Supplement: Additional file 1 — Amino acid sequence alignment of hLRRK2 and dLRRK. The data provided the amino acid sequence alignment of hLRRK2 and dLRRK. hLRRK2 amino acid sequences (NP_940980) and dLRRK amino acid sequences (CG5483, NP_650903) are from NCBI protein database. Alignment was done with MacVector™ 7.2.2. "*" Indicates identical amino acids between the two sequences. "." Indicates conserved amino acids between the two sequences. Highlighted purple indicates Roc GTPase domain. Highlighted red indicates kinase domain. Highlighted blue indicates the conserved PD associated point mutation sites between human and Drosophila. [file 1750-1326-3-3-S1.doc]

Additional file 1: Amino acid sequence alignment of *h*LRRK2 and *d*LRRK

*h*LRRK2 1 MASGSCQGCEEDEETLKKLIVRLNNVQEGKQIETLVQILEDLLVFTYSEH 50

*d*LRRK 1 MEHPKTGTETALEACDYFVDEVIEAS------SIRDAREEVRQIKHGEL 43

*.* *. . . .. *.. *

*h*LRRK2 51 ASKLFQGKNIHVPLLIVLDSYMRVASVQQVGWSLLCKLIEVCPGTMQSLM 100

*d*LRRK 44 RTAVISGDERTVRVLLAALGTERQIIVNMAPSGANTLLFLACQSGYESIT 93

. . * * .*. * *. . * * .*.

*h*LRRK2 101 G-PQDVGNDWEVLGVHQLILKMLTVHNASVNLSVIGLKTLDLLLTSGKIT 149

*d*LRRK 94 QRLLDAGADGRSHAVTKYSPLYAAVHSGHLGIARLMLDHFPELIQQPTVE 143

* * * * . .** .... . * *. .

*h*LRRK2 150 LLILDEESDIFMLIFDAMHSFPANDEVQKLGCKALHVLFERVSEEQLTEF 199

*d*LRRK 144 RWLP-------------LHAACINGHIK---------LLELLISYSYPDY 171

. .*. * .. * * . ..

*h*LRRK2 200 VENKDYMILLSASTNFKDEEEIVLHVLHCLHSLAIPCNNVEVLMSGNVRC 249

*d*LRRK 172 LYQT-----------YRDEE--------GQWEWRLPFDANAHDVTGQTSL 202

. . ..*** .* ..*.

*h*LRRK2 250 YNIVVEAMKAFPMSERIQEVSCCLLHRLTLGNFFNILVLNEVHEFVVKAV 299

*d*LRRK 203 YIASILGNKQLVGVLLKWQLHC----RRTLG------------------- 229

* . * .. * * ***

*h*LRRK2 300 QQYPENAALQISALSCLALLTETIFLNQDLEEKNENQENDDEGEEDKLFW 349

*d*LRRK 230 -----DSASSVSTPITPTRKRISFGIQAIMSKLHISGES--EGPDD---- 268

.* .*. . . . .. . . * ** .*

*h*LRRK2 350 LEACYKALTWHRKNKHVQEAACWALNNLLMYQNSLHEKIGDEDGHFPAHR 399

*d*LRRK 269 -----LASQES-----TECQRCPINVNLLCG----------------AAR 292

* . * *** * *

*h*LRRK2 400 EVMLSMLMHSSSKEVFQASANALSTLLEQNVNFRKILLSKGIHLNVLELM 449

*d*LRRK 293 ETALLAAVR-------------------------------GGHLDVVQSL 311

* * .. * ** *.. .

*h*LRRK2 450 QKHIHSPEVAESGCKMLNHLFEGSNTSLDIMAAVVPKILTVMKRHETSLP 499

*d*LRRK 312 LQHGANPNIVAK-------------------------------------- 323

.* * .

*h*LRRK2 500 VQLEALRAILHFIVPGMPEESREDTEFHHKLNMVKKQCFKNDIHKLVLAA 549

*d*LRRK 324 -----------------PVEDHNDPKCCEEIYGLSNVPIAEACKQRSLAM 356

* * . * . . .. **

*h*LRRK2 550 LNRFIGNPGIQKCGLKVISSIVHFPDALEMLSLEGAMDSVLHTLQMYPDD 599

*d*LRRK 357 LDLLLKHGARDDNGTAIGMAITCG---------DEAILSRLLARRVHPDS 397

* . . * . .* . *. * * . .. **

*h*LRRK2 600 QEIQCLGLSLIGYLITKKNVFIGTGHLLAKILVSSLYRFKDVAEIQTKGF 649

*d*LRRK 398 D-----------YKINKK---------------------GLPTPVEVNVF 415

* *.** . .. *

*h*LRRK2 650 QTILAILKLSASFSKLLVHHSFDLVIFHQMSSNIMEQKDQQFLNLCCKCF 699

*d*LRRK 416 LPSTSNISYSAMFP-----NNPTIIDWHSMGSSVQLSVVR---------- 450

. . ** * . .. * * * . .

*h*LRRK2 700 AKVAMDDYLKNVMLERACDQNNSIMVECLLLLGADANQAKEGSSLICQVC 749

*d*LRRK 451 ---------------------VPWMVSGVLLLNP---------------- 463

** .***.

*h*LRRK2 750 EKESSPKLVELLLNSGSREQDVRKALTISIGKGDSQIISLLLRRLALDVA 799

*d*LRRK 464 KLQSHPRLNEVALTAITR-IDFSHNVLTSIP----QELFHLVSLRYLNVA 508

.* *.* *. *.. .* * . . ** * . *. * **

*h*LRRK2 800 NNSICLGGFCIGKVEPSWLGPLFPDKTSNLRKQTNIASTLARMVIRYQMK 849

*d*LRRK 509 QNKITDLPAPIGQTYG-------CPVLDELFLQDNQLTTLPAAIFHLPAL 551

.* *. **. * * * .** . .

*h*LRRK2 850 SAVEEGTASGSDGNFSEDVLSKFDEWTFIPDSSMDSVFAQSDDLDSEGSE 899

*d*LRRK 552 SILDVSNN--------KLQQLPFDLWRAPKLRELNVAFNLLRDLP----- 588

* .. . ** * . * **

*h*LRRK2 900 GSFLVKKKSNSISVGEFYRDAVLQRCSPNLQRHSNSLGPIFDHEDLLKRK 949

*d*LRRK 589 ----VPPMQTSSSLLSLDKLNLQSFEEPPSNKPRNVTQQRLTHRNLWSAT 634

* .* *. . . * .. * * *

*h*LRRK2 950 RKILSSDDSLRSSKLQSHMRHSDSISSLASEREYITSLDLSANELRDIDA 999

*d*LRRK 635 LDITDND-----MKWQHEQDLGDGKTAGVGS------------------- 660

* * * * * ..

*h*LRRK2 1000 LSQKCCISVHLEHLEKLELHQNALTSFPQQLCETLKSLTHLDLHSNKFTS 1049

*d*LRRK 661 -----------SQLSSLNIANNLFTSIPAALPCLAVNLTRLNMSYNSLRS 699

.* * . .* ** * * **.* . * *

*h*LRRK2 1050 FPSYLLKMSCIANLDVSRNDIGPSVVLDPTVKCPTLKQFNLSYNQLSFVP 1099

*d*LRRK 700 MG-------------------------HVTSYPATLKQLDLSHNEISCWP 724

* **** ** *..* *

*h*LRRK2 1100 ENLTDVVEKLEQLILEGNKISGICSPLRLKELKILNLSKNHISSLSENFL 1149

*d*LRRK 725 S-LPRITESDPHLLCYS------CVQLPE------------GR--DDDYK 753

* . * .*. * * . .

*h*LRRK2 1150 EACPKVESFSARMNFLAAMPFLPPSMTILKLSQNKFSCIPEAILNLPHLR 1199

*d*LRRK 754 TASSKGSSS---------------SATSFRASVLKSVCRHRRHLRLEALR 788

*. * * * * . * * * * * **

*h*LRRK2 1200 SLDMSSNDIQYLPGPAHWKSLNLRELLFSHNQISILDLSEKAYLWSRVEK 1249

*d*LRRK 789 TLILADNLLTRIQLSTDDATTLFNE--SEDADWSVVGVNRSKVIFPNLSM 836

.* .. * . . . . * *.. . . .

*h*LRRK2 1250 LHLSHNKLKEIPPEIGCLENLTSLDVSYNLELRSFPNEMGKLSKIWDLPL 1299

*d*LRRK 837 LDMTNNCLKEIPASLHELSSLSVLNISGNVNITELPPHLGLLSRLWNLNT 886

* ...* ***** . * *. * .* *. . * .* **..* *

*h*LRRK2 1300 DELHLNFDFKHIG----CKAKDIIRFLQQRLKKAVPYNRMKLMIVGNTGS 1345

*d*LRRK 887 RGCLLQEPLRSMIESKKHKTMDIVGYLKSIYEDAQPYARMKLMVVGVAGI 936

*. . . *. **. .*. * ** *****.** .*

*h*LRRK2 1346 GKTTLLQQLMKTK----------------------------------KSD 1361

*d*LRRK 937 GKSTLLDLLRQGAGSGSSSSSHRSRASENHWAKRMGHARSTSRSHRHSSA 986

**.*** * . *

*h*LRRK2 1362 LGMQSATVGIDVKDWPIQIRDKRKR----DLVLNVWDFAGREEFYSTHPH 1407

*d*LRRK 987 SSANISTVGVDIGTWICEKR-KRAPGSHGPVVFRTWDFGGQKEYYATHQY 1035

. .***.*. * . * ** .* *** *. *.*.**

*h*LRRK2 1408 FMTQRALYLAVYDLSKGQAEVDAMKPWLFNIKARASSSPVILVGTHLDVS 1457

*d*LRRK 1036 FLSKRSLYLVLWRISDGHKGLAELLQWLGNIQARAPNSPVIIVGTHFDAV 1085

*...*.*** . .* *. . . ** **.*** ****.**** *

*h*LRRK2 1458 DE---KQRKACMSKITKELLN------KRGFPAIRDYHFVNATEESDALA 1498

*d*LRRK 1086 GESISPQKAEQLQQLIREKFIAIPDAEKIGLPRVIDSIEISCR-TLHNIH 1134

* *. . .. .* * * * . * . . .

*h*LRRK2 1499 KLRKTIINESLNFKIR--DQLVVGQLIPDCYVELEKIILSERKNVPIEF- 1545

*d*LRRK 1135 LLANIIYDTAMQLRSPGSKEPMLLQKIPASYIALEDIVNVIACNLRAAGR 1184

* * ... . . .. * ** .*. ** *. *.

*h*LRRK2 1546 -PVIDRKRLLQLVRENQ------LQLDENELPHAVHFLNESGVLLHFQDP 1588

*d*LRRK 1185 DPVLDGEQYKRLVTEQMRLHNYKSFRDAAELQQATTWCHENGVLLHYDDA 1234

**.* . .** *. * ** .* .* *****. *

*h*LRRK2 1589 ALQLSDLYFVEPKWLCKIMAQILTVKVEGCPKHPKGIISRRDVEKFLSKK 1638

*d*LRRK 1235 TLR--DYYFLDPQWLCDMLAHVVTVR-EINPFAPTGVMKLDDLQMLFRSV 1281

.*. * **..*.*** ..*...**. * * * *.. *..

*h*LRRK2 1639 RKFPKNYMSQYFKLLEKFQIALPIGEEYLLVPSSLSDHRPVIELP--HCE 1686

*d*LRRK 1282 QVQGNGNRSYIVSLLNKFEVALTWDSRTLLIPSLLPSQEAATPNSGSTVK 1331

. . * ** **..** **.** * .

*h*LRRK2 1687 NSEIIIRLYEMPYFPMGFWSRLINRLLEISPYMLSGR----------ERA 1726

*d*LRRK 1332 LSQGLRRILLMTYFPSGFWSRLITRILADEQIIEAIRGVYMASQDYADFD 1381

*. . *. * *** *******.*.* . . * .

*h*LRRK2 1727 LRP------NRMYWRQGIYLNWSP-------------------------- 1744

*d*LRRK 1382 LRTSLEQDTQWNLWQTGLALYYGPILIFKIWEVPFQKTERTQPFRTDGNR 1431

** . *. *. * *

*h*LRRK2 1745 ------------------------EAYCLVGSEVLDNHPESFLKITVPSC 1770

*d*LRRK 1432 FKLKQDGIWSDVNLSSSSILEVYFPLYEVNISQEVDDNERQLLAEIRPHM 1481

* . *. .* . * *

*h*LRRK2 1771 RKGCILLGQVVDHIDSLMEEWFPGLLEIDICG-EG----------ETLLK 1809

*d*LRRK 1482 SQVAKLLALTVDHIDLLLEDWYPSLGTRFVHTSEGRFLITRLVLCPRCLW 1531

. . ** ***** *.*.*.* * . ** *

*h*LRRK2 1810 KWALYSFND---------------------------------GEE----- 1821

*d*LRRK 1532 KLQLQQNNEPSDREVPPVGCNRPSRSSRRGAGAYFLHGVGDPGEDGALNV 1581

* * *. **.

*h*LRRK2 1822 ---------HQKILLDDLMKKAEEGDLLVNPD------------QPRLTI 1850

*d*LRRK 1582 FSAYLNATARRERRSEDSLGAGSDADSGVGPDSAGSSRNTSVDGHPGYHL 1631

.. .* . . * *.** .* .

*h*LRRK2 1851 P---------------------------------ISQIAPDLILADLPRN 1867

*d*LRRK 1632 PDNSNVCYAWMIEECILSVYNQSKISCPVHLEQSMAQLAPDVIFADIPDK 1681

* ..*.***.* **.*

*h*LRRK2 1868 IMLNNDELEFEQAPEFLLGDGSFGSVYRAAYEGEEVAVKIFN-------- 1909

*d*LRRK 1682 HTIPS----ECIIKGSLLGRGAFGFVFKANCKSALMAFKVAVGKWDRDPL 1727

. *** *.** *..* .* *.

*h*LRRK2 1910 --KHTSLRLLRQELVVLCHLHHPSLISLLAAGIRPRMLVMELASKGSLDR 1957

*d*LRRK 1728 QHSCKAYCTARQELAVLLTLKHPNIVPLVGICIKPLALVLELAPLGGLDA 1777

. **** ** *.** .. *. *.* **.*** * **

*h*LRRK2 1958 LLQQDK---ASLTRTLQHRIALHVADGLRYLHSAMIIYRDLKPHNVLLFT 2004

*d*LRRK 1778 LLRHYRRSGAHMGPHTFQTLVLQAARAIEYLHRRRIIYRDLKSENVLVWE 1827

**.. . * . . . *. * . *** ******* ***.

*h*LRRK2 2005 L-------YPNAAIIAKIADYGIAQYCCRMGIKTSEGTPGFRAPEVARGN 2047

*d*LRRK 1828 LPQPHTEDSPRNLVHIKIADYGISRQTAPSGAKGFGGTEGFMAPEIIR-- 1875

* * . *******.. .. * * ** ** ***. *

*h*LRRK2 2048 VIYNQQADVYSFGLLLYDILTTGGRIVEGLKFPNEFDELEIQGKLPDPVK 2097

*d*LRRK 1876 --YNGHESIKEC-------------ILEGSRPALTQRETQFPTCCLD--- 1907

** . . *.** . * . *

*h*LRRK2 2098 EYGCAPWPMVEKLIKQCLKENPQERPTSAQVFDILNSAELVCLTRRILLP 2147

*d*LRRK 1908 ------------LMVLCWHEQPRRRPTASQIVSILSAPECIHLLDVVAMP 1945

*. * .*.*. ***..*. ** . * . * . .*

*h*LRRK2 2148 KNVIVECMVATHHNSRNASIWLGCGHTDRGQLSFLDLNTEGYTSEEVADS 2197

*d*LRRK 1946 HSEKIVCGVFQSLVGMGDDERCGLELWLPSFGSRIDILDCSPSGSLLQCN 1995

. . * * . * * .*. . .

*h*LRRK2 2198 RILCLALVHLPVEKE---SWIVSGTQSGTLLVINTEDGKKRHTLEKMTDS 2244

*d*LRRK 1996 SISCSPQPQVAPPKTPENGANSRARSAQRLPKMNMLCCCLVGEAIWMGDV 2045

* * .. * . * .* * *

*h*LRRK2 2245 VTCLYCNSFSKQSKQKNFLLVG---TADGKLAIFEDKTVKLKGAAPLKIL 2291

*d*LRRK 2046 SGNLHAYSTSTYAHLFSYMLDPNIKSAVISLVYMEKIARVAVGTHNGRVF 2095

* . * * .. ..* .* * * . *. ..

*h*LRRK2 2292 NIGNVSTPLMCLSES-----TNSTERNVMWGGCGTKIFSFSNDFTIQKLI 2336

*d*LRRK 2096 LVDATQMPSNCAFAEGSFVLTEICSGFVLHAACSVVVDGIYELWCGEIAG 2145

. * * * . *. * . . .

*h*LRRK2 2337 ETRTSQLFS-----YAAFSDSNIITVVVDTALYIAKQNS--------P-- 2371

*d*LRRK 2146 KINVFPLNENGVSGHQALCHSEEPNLIEDVKVARMCSNESHVFSCLYPGC 2195

* * . * ... * . * *

*h*LRRK2 2372 VVEVWDKKTEKLCGLIDCVHFLREVMVKENKESKHKMSYSGRVKTLCLQK 2421

*d*LRRK 2196 MVYQWDVISKRIENKLDCSKLLPCSESLQSIAIDEHVNLIKCQISALAAH 2245

.* ** . .. . .** . * . .. . .

*h*LRRK2 2422 NTALWIGTGGGHILLLDLSTRRLIRVIYNFCNSVRVMMTAQLGSLKNVML 2471

*d*LRRK 2246 NSELYIGTTWGCLIVAELHTLRPISVFRPYENEIKSIITLSK---DNVPL 2292

*. * *** * ... .* * * * * . * .. ..* ** *

*h*LRRK2 2472 VLGYNRKN-TEGTQKQKEIQSCLTVWDINLP-HEVQNLEKHIEVRKELAE 2519

*d*LRRK 2293 IATIGRRYRSLISRYVDSAESSTKSSAVSTPTHGAAKSVPPADVDNHIHC 2342

. .*. . .. .*. . * * .* .

*h*LRRK2 2520 KMRRTSVE 2527

*d*LRRK 2343 LLWRAKHWT 2351
